# Robot-assisted stereotactic brainstem biopsy in children: prospective cohort study

**DOI:** 10.1007/s11701-018-0899-x

**Published:** 2018-12-06

**Authors:** William Dawes, Hani J. Marcus, Martin Tisdall, Kristian Aquilina

**Affiliations:** 1 Department of Neurosurgery, Great Ormond Street Hospital, London, UK; 2 Wellcome EPSRC Centre for Interventional and Surgical Sciences, University College London, 8.02 Malet Place Building, Gower Street, London, WC1E 6BT UK

**Keywords:** Surgery, Robotics, Stereotaxy, Diffuse intrinsic brainstem glioma, DIPG

## Abstract

Tumours located within the brainstem comprise approximately a tenth of all paediatric brain tumours. Surgical biopsy of these tumours is technically challenging and has historically been associated with considerable risk. To this end, robot-assisted surgery theoretically allows for increased accuracy and precision. In this study we report our experience using the Neuromate robot (Renishaw, Gloucestershire, UK) to perform robot-assisted stereotactic biopsy in children with tumours located within the brainstem. An uncontrolled prospective cohort study was performed (phase II) according to the IDEAL model for safe surgical innovation. All cases were recorded on a prospectively maintained database. The database was searched over a 2-year period between the 1st December 2015 and the 31st November 2017 to identify all children with brainstem tumours that underwent robot-assisted stereotactic brain biopsy. When accessible, the post-operative MRI scans and pre-operative plans were compared to assess the target point localisation error (TPLE). Adverse events were recorded prospectively according to whether they resulted in increased hospital stay, caused neurological injury, or lead to death. In all, 11 consecutive children were identified with brain tumours located within the brainstem. In 10/11 cases specimens were diagnostic; in the remaining case a further biopsy was successful. The most frequent pathology was DIPG (7/15). Seven patients underwent an early post-operative volumetric MRI; the calculated median TPLE was 2.7 mm (range 0.5–4.2 mm). There were no surgical complications noted. Robot-assisted stereotactic biopsy in children appears to be feasible and safe. Research databases and comparative studies are warranted to further assess the technique.

## Introduction

Tumours located within the brainstem comprise approximately a tenth of all paediatric brain tumours [1]. The most common of these tumours is diffuse intrinsic pontine glioma (DIPG). As DIPG is associated with a characteristic MRI appearance and can be diagnosed from imaging alone, it has been argued that brainstem biopsy is not warranted in most cases [2]. In addition, the majority of children with DIPG die within 2 years of diagnosis, and information obtained from biopsy has, up to now, not altered treatment strategy [3]. However, recent studies have suggested that molecular markers may aid prognostication [4]. Several clinical trials that utilise these molecular markers to personalise treatment regimens are now underway. The BIOMEDE trial (NCT02233049), for example, specifically randomises treatment to targets identified on biopsy, and importantly withholds treatment to targets that are not identified. A prerequisite to the enrolment of patients into such clinical trials is the acquisition of brain tumour tissue.

Surgical biopsy of brainstem tumours is technically challenging and has historically been associated with considerable risk [2]. Stereotactic brain biopsy is generally preferred to open biopsy unless an exophytic tumour component is identified. The most common trajectories are transcortical (transfrontal) via the cerebral peduncle and transcerebellar via the middle cerebellar peduncle; the former requires a longer trajectory and carries a risk of injury to the ventricles, while the latter is performed prone and necessitates dissection of the nuchal musculature that is associated with increased pain. Whichever approach is selected, high accuracy and precision is mandated; if these requirements are met, a number of publications have demonstrated that the risks of brainstem tumour biopsy are potentially low [5–7].

Robot-assisted surgery theoretically allows for increased accuracy and precision. The first report of a robot-assisted stereotactic brain biopsy was in 1985 when a modified PUMA industrial robot (Advance Research and Robotics, CT, USA) was used to define the trajectory [8]. Although several subsequent studies have concluded that robot-assisted stereotactic brain biopsy is feasible and safe [8–13], few have focused on children with brainstem pathology, who represent a unique patient group.

In this study, we report our experience using the Neuromate robot (Renishaw, Gloucestershire, UK) to perform robot-assisted stereotactic biopsy in children with tumours located within the brainstem.

## Methods

The study was registered as a Service Evaluation study with the Great Ormond Street Hospital for Children NHS Foundation Trust Clinical Audit Committee (#2237). Informed consent was not sought, as this was a Service Evaluation study.

An uncontrolled prospective cohort study was performed (phase II) according to the IDEAL model for safe surgical innovation. The Strengthening the Reporting of Observational Studies in Epidemiology (STROBE) Statement was used in the preparation of this section of the manuscript.

### Setting and participants

The study was conducted at Great Ormond Street Hospital, the regional referral centre for paediatric tumours in North London. Two senior neurosurgeons (MT and KA) performed all robot-assisted procedures.

All cases were recorded on a prospectively maintained database. The database was searched over a 2-year period between the 1st December 2015 and the 31st November 2017 to identify all children with deep-seated lesions that underwent robot-assisted stereotactic brain biopsy.

### Variables and data sources

Patients had a pre-operative volumetric MRI with contrast to identify the tumour and the optimal location for biopsy was determined using a multidisciplinary approach. Images were then transferred to the Neuromate robot and an entry point (EP) and target point (TP) used to define a safe surgical trajectory that avoided eloquent tissue, vasculature, and transependymal passage. Patients were then placed under general anaesthesia, a Leksell frame placed, and a CT head performed; this was merged with the pre-operative MRI scan depending on whether a transcortical or transcerebellar approach was used, patients were positioned supine or prone, respectively, and the robot docked. Once registration was performed the robot aligned the tool holder along the vector of the planned trajectory; a stereotactic biopsy using a side-cutting Sedan needle was then performed in the usual fashion.

Specimens were sent fresh for definitive histopathological analysis. A biopsy was considered positive if specimens resulted in a diagnosis based on histology or molecular markers.

**Fig. 1:**
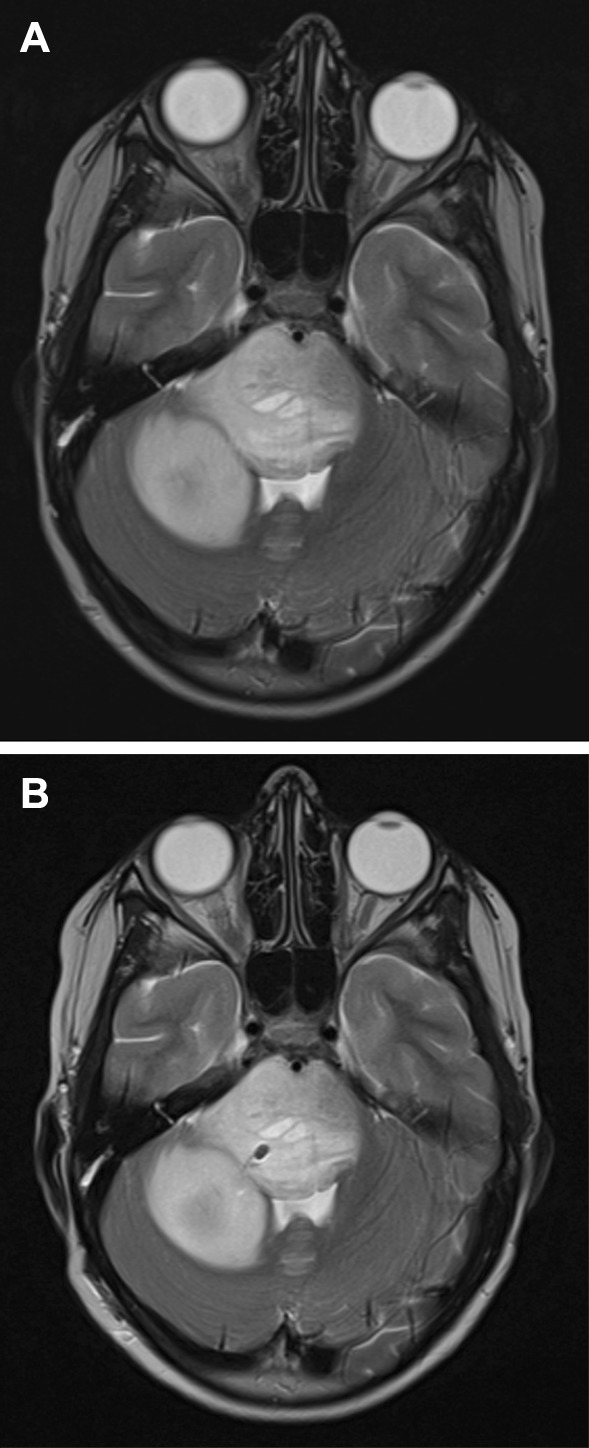
**a** Pre-operative and **b** post-operative axial T2-weighted MRI scan of demonstrating pontine lesion and right-sided transcerebellar approach

**Fig. 2:**
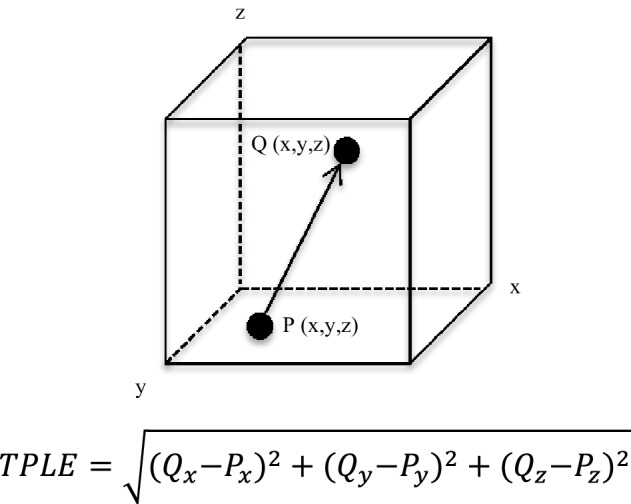
Target point localisation error (TPLE) is defined as the Euclidian distance between the planned and actual target. The planned biopsy target is represented by point *P*, and the actual biopsy target by point *Q*. The arrow is the Euclidian distance between these two points

A post-operative volumetric CT or MRI was performed in selected cases depending on their availability and the surgeon’s preference. When accessible, the post-operative T2-weighted MRI scan and pre-operative plan were loaded on the Neuromate robot and compared by a surgeon (HJM) to assess the target point localisation error (TPLE) (Fig. 1). The TPLE is defined as the Euclidian distance between the planned and actual target (the centre of the biopsy cavity), and is calculated by determining the square root of the sum of the squares of the differences between these co-ordinates (*x, y*, and *z*) (Fig. 2).

Patients were followed up approximately 2 weeks after surgery for wound review and subsequently transferred to the care of oncology unless on-going concern. Adverse events were defined as any untoward event related to a child’s admission that led to an increase in hospital stay, caused neurological injury, or led to death. Adverse events within 30 days of surgery were graded and recorded prospectively in the following fashion: (1) no increase in hospital stay and no neurological injury; (2) increase in hospital stay but no neurological injury; (3) neurological injury, and (4) death. The strength of this classification system is that it records all adverse events whether or not they may be related to surgical complications.

### Study size and statistical methods

The sample size was determined on a constraint-based pragmatic approach as robot-assisted stereotactic brainstem biopsies are rare. We considered a minimum of six patients sufficient for meaningful analysis.

Data were analysed using with SPSS v 20.0 (IBM, IL, USA). The mean and standard deviation were calculated for parametric variables, and the median and interquartile ranges calculated for non-parametric variables.

## Results

### Participants and descriptive data

In all, 11 consecutive children were identified with brain tumours located within the brainstem and related structures that underwent 12 robot-assisted stereotactic brain biopsies. The patient demographics are detailed in Table 1. The median age was 10 years (range 2–15 years), and the male:female ratio was 1:1.2. Brain tumours were most commonly located in the pons (9/11) and typically a transcerebellar approach was used (9/11).

**Table 1:** Patient demographics and pathology

Case	Age	Sex	Location	Approach	Pathology
1	15	M	Pons	Right transcerebellar	Inflammatory
2	12	F	Pons	Right transcerebellar	Glioblastoma
3	11	M	Midbrain	Right transcortical	Astrocytoma
4	2	F	Pons	Right transcerebellar	DIPG
5	6	F	Midbrain	Right transcortical	Astrocytoma
6	8	M	Pons	Right transcerebellar	DIPG and radiation necrosis
7	13	F	Pons	Left transcerebellar	DIPG
8	13	M	Pons	Right transcerebellar	DIPG
9	5	F	Pons	Left transcerebellar	DIPG
10	9	F	Pons	Left transcerebellar	DIPG
11	10	M	Pons	Right transcerebellar	DIPG

### Outcome data and main results

In 10/11 cases specimens were diagnostic; in the remaining case a further biopsy was successful. The patient pathologies are detailed in Table 1. The most frequent pathology was DIPG (7/11).

Seven patients underwent an early post-operative volumetric MRI. The calculated median TPLE was 2.7 mm (range 0.5–4.2 mm).

There were no surgical complications noted. The median length of stay was 2 days (range 2–8 days).

## Discussion

### Principal findings

We found that robot-assisted stereotactic brainstem biopsy in children was both feasible and safe. In our prospective cohort study, the technique was found to be accurate (median TPLE 2.7 mm) and provide a high diagnostic yield (10/11 cases). Moreover, no significant adverse events were recorded.

### Comparison with other studies

The literature supporting the use of surgical robotics within neurosurgery is small but rapidly growing [14]. Approximately 300 robot-assisted stereotactic brain biopsy procedures have been reported in the literature over the last 30 years, but the majority have been reported within just the last 5 years [15–22].

Several studies have reported the accuracy of robot-assisted brain biopsy, albeit in adults. The average target accuracy varies in these studies from 0.9 to 4.5 mm, and is therefore comparable to our own cohort [10, 11, 23, 24].

In the largest paediatric series to date, De Benedictis et al. used the ROSA robot (Medtech, France) and performed 128 robot-assisted surgeries on 115 consecutive children, including 26 stereotactic brain biopsy procedures [17]. Although no formal TPLE was calculated, the authors reported the technique was accurate with a tissue diagnosis in 25/26 cases. One patient had transient worsening of their neurological symptoms, but otherwise no complications were noted.

LeFranc et al. used the ROSA robot and performed robot-assisted stereotactic biopsy in 100 patients, including several children (median age 59 years; range 7–86 years) [18]. A tissue diagnosis was made in 97/100 cases. Six patients were found to have a post-operative haematoma on imaging, which were associated in transient worsening of neurological symptoms in two cases. Four further patients also had a transient worsening of their neurological symptoms.

Other studies using the ROSA robot have also found robot-assisted stereotactic biopsy in children to be feasible and safe. Miller et al. reported successful biopsy in six patients, Quick-Weller et al. in two patients, Carai et al. in seven patients, and Coca et al. in five patients [16, 19–21]; a tissue diagnosis was made in 20/20 cases, and transient worsening of neurological symptoms in only one patient.

Haegelen et al. used the Neuromate robot and performed robot-assisted stereotactic brainstem biopsy in 15 patients, including 5 children [15]. A tissue diagnosis was made in 13/15 cases. One case had permanent post-operative morbidity and two others transient morbidity.

A possible confounder when interpreting the aforementioned studies, is their use of either the transcortical or transcerebellar approach, which is frequently not reported. However, in a retrospective study Dellaretti et al. compared these approaches and found no significant difference in diagnostic yield or safety [13].

### Limitations

The present study has several limitations. Unfortunately, the operative time, which has been raised as a concern with robot-assisted surgery, was not prospectively recorded. Also, early post-operative imaging was only available in selected patients limiting the assessment of accuracy.

More generally, the sample size was small as robot-assisted stereotactic brainstem biopsies are rare. Lastly, the uncontrolled prospective cohort study design does not allow for rigorous comparison between robotic and standard stereotactic brain biopsy.

## Conclusion

Robot-assisted stereotactic brainstem biopsy in children appears to be both feasible and safe. With rapid advances in robotics, it is likely that future platforms will become smaller, more powerful, and less costly, in a way analogous to digital computing over the last half century [25]. Eventually, a tipping point will be reached, allowing for their more widespread dissemination. To this end, research databases and comparative studies are now warranted to further assess the technique in line with the IDEAL model for safe surgical innovation.
